# Temporal and spatial aggregation of rainfall extremes over India under anthropogenic warming

**DOI:** 10.1038/s41598-024-63417-w

**Published:** 2024-05-31

**Authors:** Gopinadh Konda, Jasti S. Chowdary, C. Gnanaseelan, Naresh Krishna Vissa, Anant Parekh

**Affiliations:** 1 Indian Institute of Tropical Meteorology, Ministry of Earth Sciences, Pune, 411008 India; 2 Department of Earth and Atmospheric Sciences, National Institute of Technology, Rourkela, 769008 India

**Keywords:** Atmospheric science, Climate sciences

## Abstract

India experienced several unprecedented floods in the recent decades. The increase in the extreme rainfall events (EREs) is the primary cause for these floods, manifesting its societal impacts. The daily downscaled and bias corrected (DBC) Coupled Model Intercomparison Project Phase 6 (CMIP6) rainfall and sea surface temperature (SST) are prepared for the Indian region and are utilized to examine the characteristics of EREs. The DBC products capture the characteristic features of EREs for the baseline period, which inspired us to assess the EREs over India in CMIP6 future projections. Consistent with the observations, DBC product shows ~ 8% of Indian land found to experienced extremely heavy rainfall associated with the long duration EREs in the baseline period. However, area and extreme rainfall thresholds are projected to increase by about 18(13)% and 58(50)%, respectively in the far future under SSP5-8.5 (SSP2-4.5) emission scenario relative to the baseline period. A two-fold-65(62)% increase in long-duration EREs compared to the short-duration EREs and substantial warming ~ 2.4(2.9) ^o^C of Indian Ocean SSTs in the far future under SSP5-8.5 (SSP2-4.5) emission scenario compared to baseline period are reported. These findings may provide fundamental insights to formulate national climate change adaptation policies for the EREs.

## Introduction

Understanding, predicting and projecting the extreme events have become subjects of prime importance and concern for scientists considering their social, economic, and environmental impacts^1,2^. Extreme events caused by natural hazards or/and human actions may, in turn, trigger natural and technological disasters^3^. Variations in the intensity and distribution of extreme rainfall patterns are important due to the possible threat to human activities posed by extreme precipitation and floods. Climate extremes demand preparedness and emergency response strategies for different sectors including health and social care providers^4^. A recent study also confirmed that extreme rainfall reduces worldwide macroeconomic growth rates and slows down the progress of the global economy^5^. The CO_2_-induced climate change, which has already started impacting different areas around the globe, is expected to be more threatening in future decades and will have long-term implications in many sectors.

The South Asian summer monsoon rainfall from June to September plays a critical role in providing food for almost one-half of the world's population. The rainfall variability makes this region one of the most vulnerable areas worldwide and impacts climate-related natural disasters such as droughts and floods^6^. In recent decades, Indian summer monsoon (ISM) rainfall has been amplified through the modulation of monsoon circulation^7–9^, and a significant rise in extreme rainfall events (EREs)^10–14^. An increase in greenhouse gases leads to increased intensity and frequency of EREs over the globe^15^, and the increased water vapour leads to more intense precipitation events^16–18^. Using high-resolution data, previous studies pointed out that the increase in magnitude and frequency of EREs over central India during monsoon season is due to an increase in the moisture transport from the adjacent oceans and the rapid warming of the equatorial Indian Ocean^10,14,19,20^. Studies also found a considerable increase in future rainfall during the summer monsoons season in central India^14^. Increased EREs over central India are attributed mainly to the propagation of monsoon depressions from the Bay of Bengal (BoB) with a life span of 3–6 days, as they carry more moisture into central India^21–23^.

Both climate model simulations and observations ascribed the rise of EREs to warming of climate^10,24–27^. Warm climate increases the moisture holding capacity of the atmosphere^28^ as explained by the Clausius-Clapeyron (C–C) relationship^26,28–30^. Recent studies also reported differences in the responses of regional monsoons to climate change due to distinct regional land-sea contrast, different feedback mechanisms, and topography^31–33^. Nevertheless, future projections of global and regional monsoons require more careful investigation^17,34^. General Circulation models (GCMs) have been widely used to examine the past and future changes in climate, and a comprehensive assessment of GCM performance is of great importance for selecting the best models for regional climate. It is well-known that one of the primary sources of uncertainty in estimating climate projections (especially in the case of extreme events) is the choice of GCM. The Coupled Model Intercomparison Project Phase 6 (CMIP6) model future projections consider the precise problems in societal development indicators, such as urbanization and economy, thus named Shared Socioeconomic Pathways (SSP)^35^. These SSPs are focused on radiative forcing ranging from low to high-emission scenarios. Recent studies found a better representation of regional features of the earth system in CMIP6 models than in earlier versions, such as CMIP5 models^36,37^. The CMIP6 models also show significant improvements in simulating rainfall extremes such as wet and dry days over the Indian subcontinent compared to the previous CMIPs^36^. The large-scale circulations associated with ISM are found to be well represented in CMIP6 models^38^. The CMIP6 model simulations show notable progress in capturing ISM rainfall’s interannual and multi-decadal variability from 1901 to 2015^39^. It has been reported that the frequency and intensity of extreme precipitation are increasing with spatial variation and is expected to continue further in the global warming^40^.

Very few studies have investigated the projected changes in rainfall extremes on a regional scale. Therefore, it is essential to examine the extremes in precipitation projections and the relative contribution of each uncertainty to the total on a regional scale. To minimize the uncertainty of the resulting GCM simulations, several studies downscaled and bias-corrected the GCM outputs at a high resolution^41^. In the present study, we downscaled and bias-corrected the precipitation and sea surface temperature (SST) products from the CMIP6 group of models. In this paper, we also assessed the ability of CMIP6 models in representing the spatio-temporal distribution of the high-impact precipitation events and its associated SST variability for the ISM season. We further estimated the possible future changes in the spatio-temporal distribution of high-impact long duration and short duration extreme rainfall events under medium and high-emission scenarios [i.e., SSP2-4.5 (SSP245) and SSP5-8.5 (SSP585)] for the period of 2015–2100. Though several previous studies reported significant trends in precipitation and EREs over India, this is the first attempt to analyze the intensity and distribution of precipitation associated with the EREs depending upon the number of occurrences and duration. An attempt is also made in this study to estimate the future changes and variability of SST associated with the EREs.

## Results

### Mean state of monsoon rainfall and SST

The seasonal mean rainfall for historical and future projections over India during the summer monsoon is shown in Fig. S1. Before DBC (downscaled and bias-corrected), multi-model-mean underestimated spatial distribution and magnitude of the ISM rainfall over India by 32%. However, after DBC, the mean ISM rainfall over India improved by about 28%, which is closer to the observations. The mean ISM rainfall over India is about 7 mm/day as indicated in the observations^42^; however, MMM before DBC (referred as MMM hereafter) the mean rainfall over India is estimated to be about 5.3 mm/day. The MMM after the DBC (referred as MMM-bc hereafter) shows a mean rainfall of about 6.8 mm/day, which is closer to the observed mean rainfall. This suggests that the MMM-bc well captured the mean rainfall distribution over homogeneous regions of India. Root mean square error and pattern correlation analysis also show significant improvement in mean rainfall over India after DBC. Future projections of CMIP6 MMM show an increase in rainfall of about 7.5% compared to the baseline period under the SSP245 scenario, whereas the increment is about 9.4% in the SSP585 scenario for the near future. However, in the far future, MMM shows an increment of 9.5% and 24% of mean ISM rainfall over India during SSP245 and SSP585, respectively. The MMM-bc product shows an increase in ISM rainfall over India by about 14% and 20% in the near future for SSP245 and SSP585 scenarios compared to the baseline period. In the far future, MMM-bc product shows 18% and 35% increment in rainfall over India under SSP245 and SSP585 scenarios, respectively. Analysis revealed that the mean ISM rainfall is projected to increase over the monsoon core region, eastern India, and Western Ghats in MMM-bc under high emission scenarios. The mean bias of ISM rainfall for the individual models before and after DBC is shown in Fig. S2. Strong dry precipitation biases are evident over the monsoon trough region in the CMIP6 models for the baseline period before the DBC; however, the INM-CM4-8 and INM-CM5-0 models show wet biases. The majority of the models overestimate the precipitation over southern peninsular India^43^. The model simulated wet and dry biases are significantly reduced over the Indian region after DBC.

The seasonal mean SST bias over the Indian Ocean for the individual CMIP6 models and the MMM for the baseline period is shown in Fig. S3. The majority of the CMIP6 models before DBC overestimate the SST over the western Indian Ocean due to the underestimation of low-level winds and the associated weakening of oceanic upwelling along the west coast of India^43,44^. GFDL-CM4 and MPI group of models strongly underestimate the SST over the Indian Ocean. After DBC, SST clearly shows reduced biases over most of the Indian Ocean. ACCESS, Earth group of models show positive SST biases across the west coast of India. The skill of the individual models and MMM in representing the ISM mean rainfall (over India) and SST (over the Indian Ocean) is shown in the Taylor-metric (Fig. S4). Compared to the models' simulated rainfall and SST before DBC, the skill with a high pattern correlation of rainfall and SST is seen after DBC. ACCESS-ESM1-5 and FGOALS-g3 models in fact failed to simulate the spatial distribution and intensity of ISM rainfall over India (Fig. S4a), whereas after DBC, these features of the ISM rainfall are well captured (Fig. S4b). MPI-ESM1-2-HR model failed to simulate the spatial distribution and intensity of SST of the Indian Ocean. However, the DBC product well captured the distribution and intensity of SST. Further, before DBC, most models display a slight underestimation of the standard deviation of rainfall and SST and after DBC they show improvement in standard deviation (close to 1).

The intensity and frequency of precipitation (over India) and SST (over the Indian Ocean; 40° to 120^o^E, 20^o^S to 30^o^N) before and after DBC are shown for historical and future projections in Fig. S5. In the historical period, the scaling rates match closely with the observations (7 mm/day) after DBC (6 mm/day). However, before DBC, MMM showed the peak intensity of precipitation at 3 mm/day and significantly underestimated the magnitude of scaling rates in both SSP245 and SSP585 scenarios compared to the baseline period. The MMM-bc product of SSP245 and SSP585 well captured precipitation’s spatial and scaling rates over India and shows good agreement with the distribution of IMD-derived scaling rates (Fig. S5a). SST scaling rates for the baseline period, SSP245 and SSP585, are shown in Fig. S5b. In the observations, peak SST is observed over ~ 28.5 °C, whereas MMM shows the peak at ~ 27.7 °C, and MMM-bc shows the peak at ~ 28.5 °C. This analysis suggests that after DBC, the representation of scaling rates of SSTs over the Indian Ocean is more realistic. Compared to the baseline period, models underestimate the scaling rates of SSTs over the Indian Ocean; however, after DBC, SST scaling rates are improved and consistent with the increasing trend of SSTs under global warming scenarios^45,46^. Overall, we can conclude that DBC precipitation and SST datasets perform well in capturing the scaling rates during monsoon season over the India/Indian Ocean region. Moreover, under high emission scenarios, precipitation and SST show increased magnitudes persisting for long period of time.

### Spatial and temporal variability of EREs

**Figure 1:**
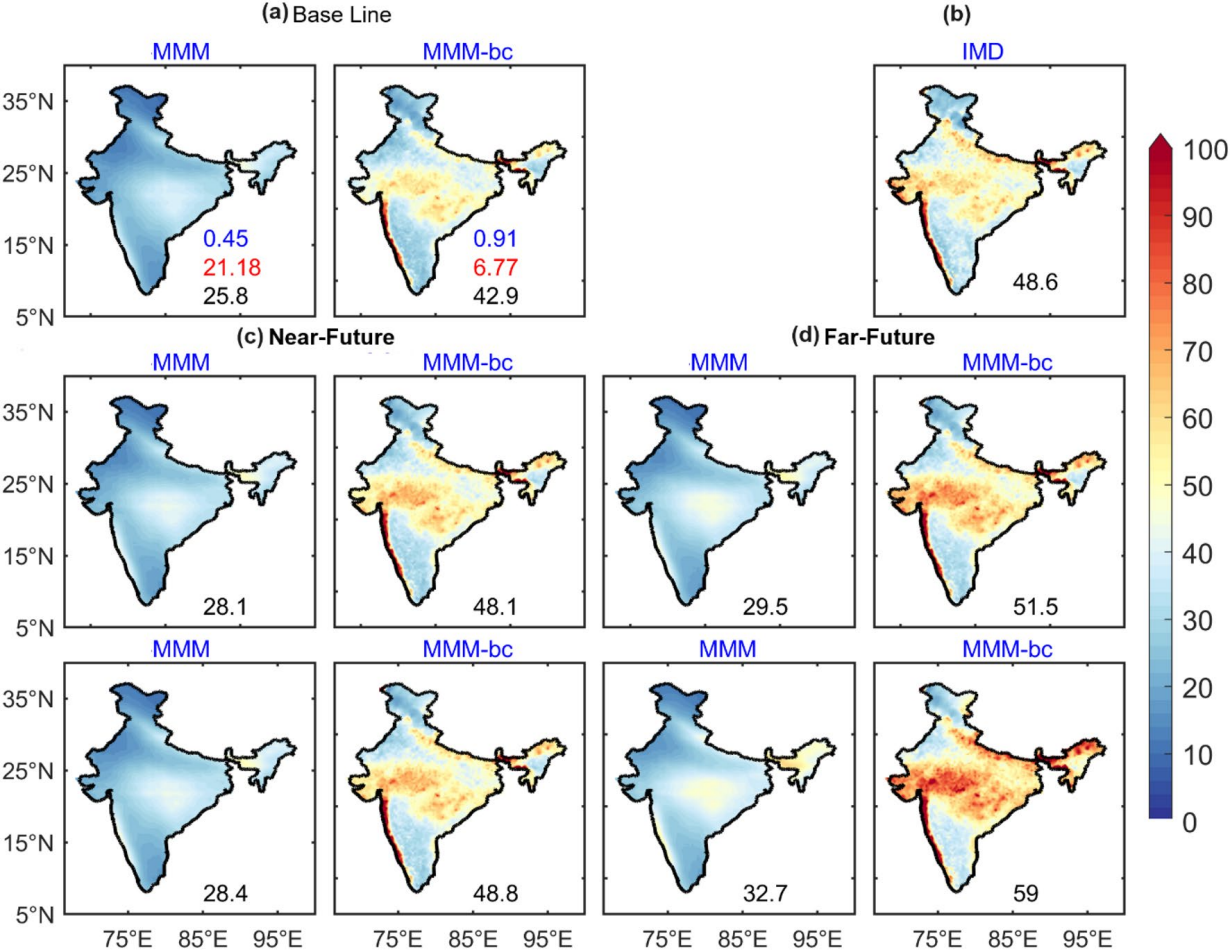
Multi model mean of Indian summer monsoon (JJAS) R95 thresholds (mm/day) over India for the historical, SSP2-4.5 (SSP245), and SSP5-8.5 (SSP585) scenarios varying from baseline (**a**), observations (IMD) (**b**), near-future (**c**), and far-future (**d**). Before DBC (MMM) and after DBC (MMM-bc). Values in blue (red) color represents the pattern correlation (RMSE). Values in black color represents the mean thresholds rainfall (mm/day). (Figure created using the Matlab R2023a; https://in.mathworks.com/).

Previous studies have analaysed the trends in precipitation and EREs in India. Nevertheless, the intensity, distribution and areal extent of precipitation associated with EREs depending on their duration in future scenarios have not been examined. The extreme threshold of precipitation (R95) over India for observations and models are shown in Fig. 1. In the observations (IMD) during the baseline period, R95 thresholds greater than 60 mm/day is located over the Western Ghats, central India, and foothills of the Himalayas, whereas models strongly underestimated the R95 thresholds over India before DBC. On the other hand, R95 thresholds improved significantly after DBC and MMM-bc product is well comparable with the observations. The mean of the R95 values over India is ~ 48.6 mm/day according to IMD rainfall and it is ~ 25.8 mm/day in the MMM of models. The mean R95 value significantly improved by 66% after DBC, and value reached to 42.9 mm/day in MMM-bc. A slight increase of R95 values is seen in the far future compared to the near future under SSP245 and SSP585 scenarios after DBC. Significant improvements in the R95 threshold are observed in MMM-bc in the historical simulations. Compared to the baseline period, the thresholds of R95 values are high during near and far-future under SSP245 and SSP585 scenarios, respectively. In the far future, the extreme threshold of precipitation would increase by 6% and 21% under the SSP245 and SSP585 scenarios, respectively. The spatial distribution of the R95 thresholds of each model is shown in Fig. S6. It is noted that the models have underestimated the magnitude of the R95 threshold over India before DBC. In fact, after DBC, significant improvements are seen in the distribution and intensity of R95. The percentage change of R95 thresholds over India in the near and far future is shown in Fig. S7. It is found that, compared to the baseline period, the MMM-bc product shows an increase of R95 thresholds by ~ 47% in the near future under SSP245 and SSP585 scenarios; however, in the far future MMM-bc product, the increase of extreme thresholds is more than 50%. The percentage change of extreme thresholds is high over the regions of west and east India, foothills of the Himalayan mountain regions (Fig. S7).

**Figure 2:**
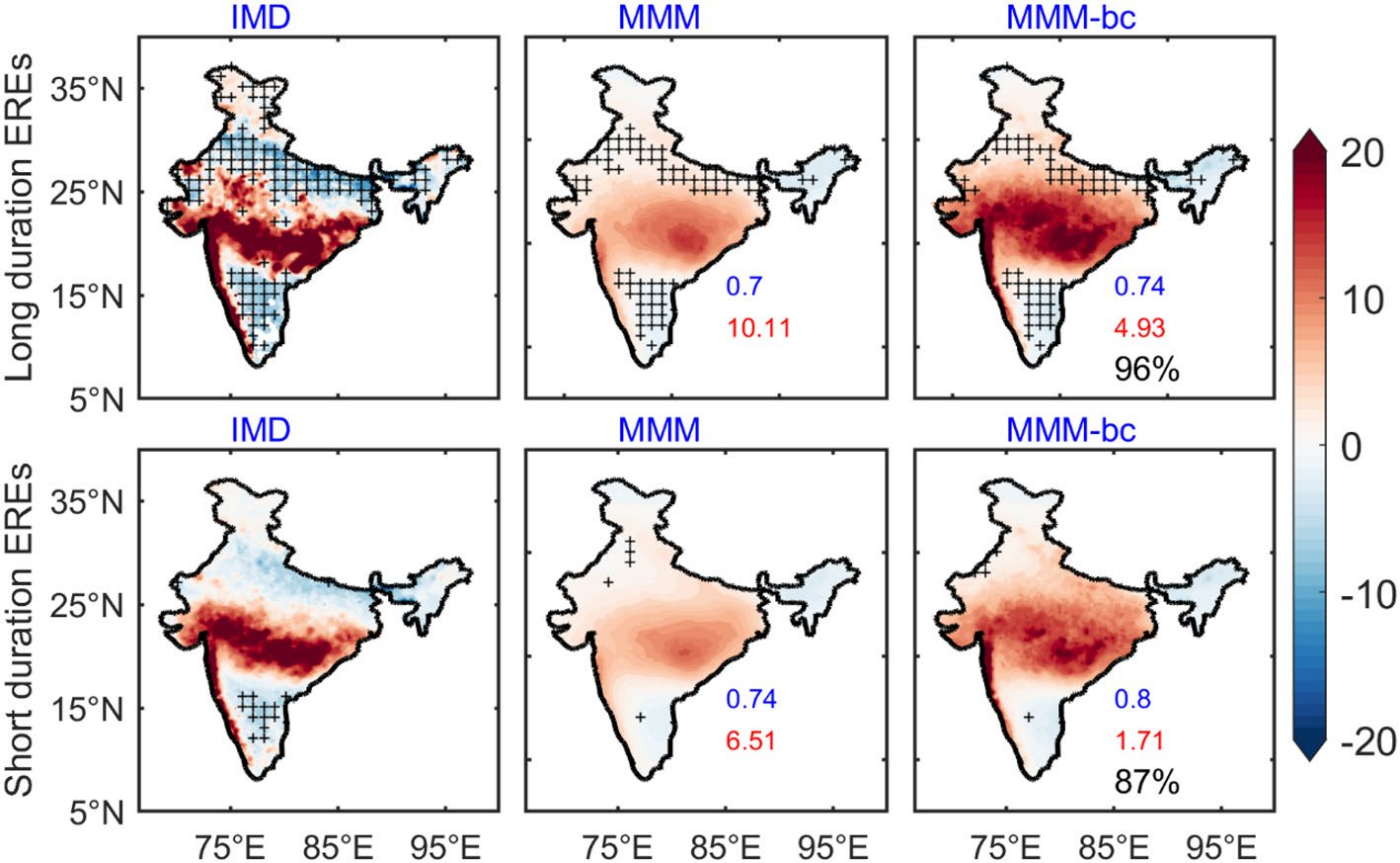
Composite of precipitation anomalies for the base-line period during long duration and short duration of EREs over India in observations (IMD) before DBC (MMM), and after DBC (MMM-bc). Values in blue (red) color represents the pattern correlation (RMSE). Improvement of mean rainfall after DBC is shown in %. Insignificant (< 95% confidence level) regions are masked with “ + ”. (Figure created using the Matlab R2023a; https://in.mathworks.com/).

**Figure 3:**
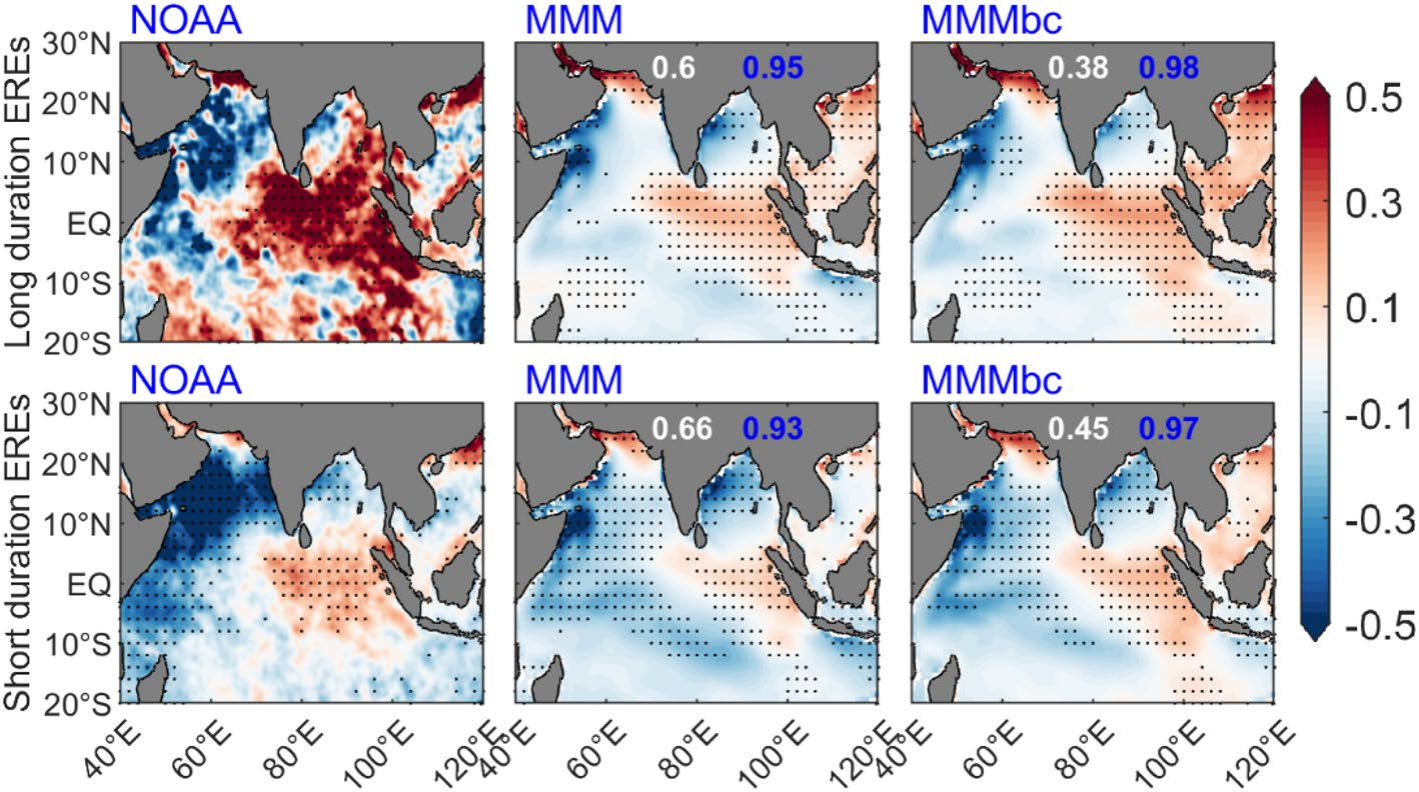
Composite of SST anomalies for the base-line period corresponds to long duration and short duration of EREs over India, for observations (NOAA), before DBC (MMM), and after DBC (MMM-bc). Values in blue (white) color represents the pattern correlation (RMSE). Significant (> 95% confidence level) regions are masked with “.”. (Figure created using the Matlab R2023a; https://in.mathworks.com/).

In terms of intensity, precipitation EREs has been classified as Low, Medium, and Heavy events^22,44,47^. Earlier studies have analyzed the spatiotemporal characteristics of EREs over the Northwest Himalaya region for the period 1998–2013^45,46^. Few studies examined the variability of EREs over India in the global warming scenario. In this work, we have studied the variability of long-duration EREs and short-duration EREs over India using the DBC product. The composite of precipitation anomalies for long-duration EREs and short-duration EREs for the baseline period is shown in Fig. 2. The composite of long-duration EREs shows high precipitation over the monsoon trough region (west to east India) and Western Ghats in the observations. Before DBC, models simulated rainfall associated with long-duration EREs is mainly found over the central eastern India region. After DBC, widespread rain is seen across the Indian land region. It is also found that the intensity and spatial distribution are improved by 96% after DBC. The analysis of short-duration EREs clearly suggests that intense precipitation is limited to central India as indicated by the observations; and after DBC, the rainfall distribution is analogous to the observations. We found 87% improvement in rainfall distribution and intensity after DBC. As compared to short duration EREs, high precipitation is observed during long-duration EREs and this characteristic features are well captured in the DBC product. It is found that precipitation intensity and spatial distribution in models/MMM-bc are well characterized similar to the observations. Estimating the impact of SST on EREs is essential to implement mitigation and adaptation options within a sustainable development framework. This study estimates the variability of SST changes associated with the EREs in India. The composite of SST during long-duration EREs shows cooling over the west Indian Ocean (WIO) and north BoB and warming over the east equatorial Indian Ocean (EEIO) in the observations (NOAA) for the historical period (Fig. 3). However, the SST composite of MMM shows weak positive SST anomalies in the EEIO, whereas in MMM-bc, SST anomalies in the EEIO intensified. In contrast, a significant reduction of cooling in the west Indian Ocean and EEIO is seen in the observations during short-duration EREs (Fig. 3). The composite of SST anomalies from MMM-bc are consistent with the observations.

**Figure 4:**
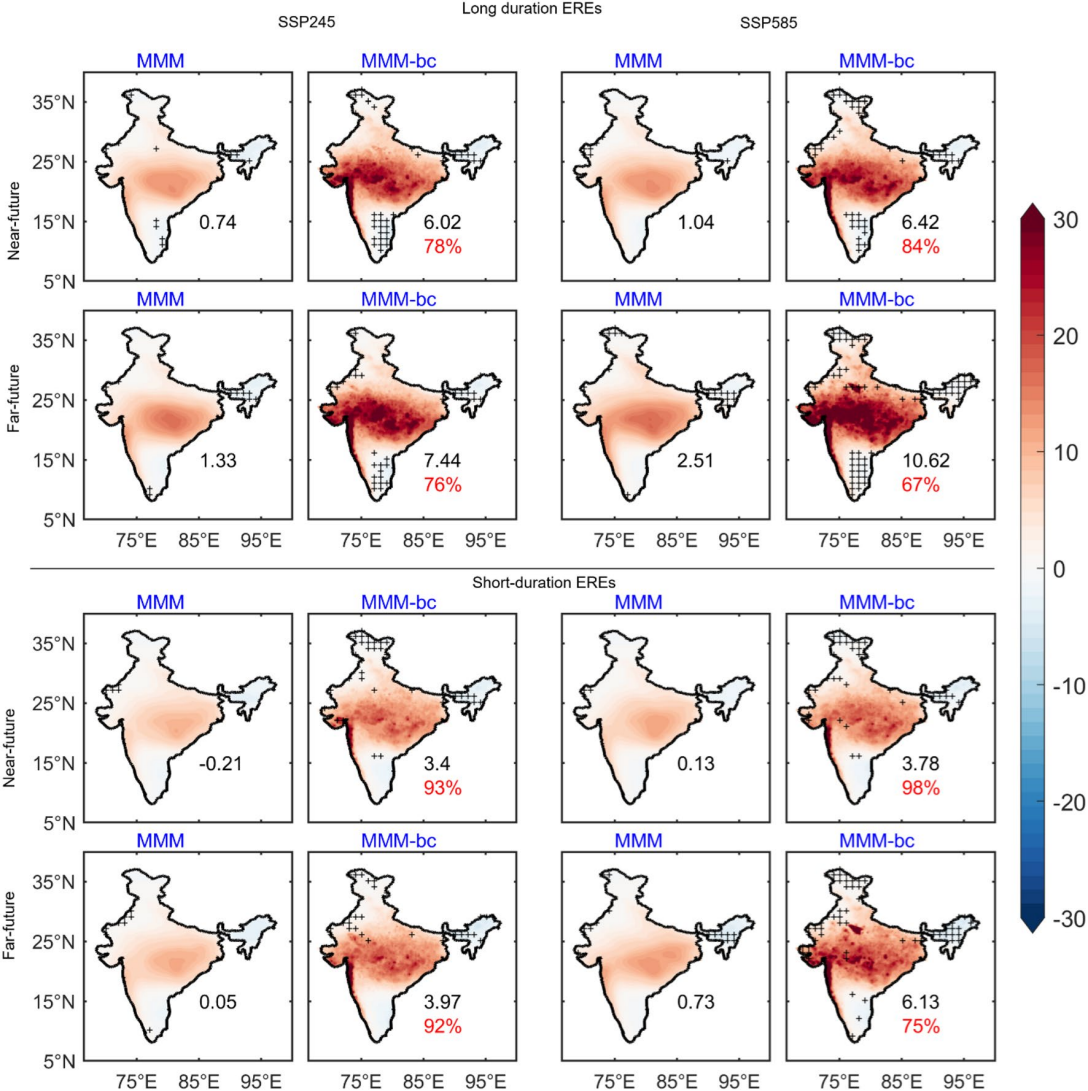
Composite of precipitation anomalies (w.r.to baseline period climatology) during long duration and short duration of EREs for SSP2-4.5 (SSP245), and SSP5-8.5 (SSP585) scenarios during near future and far future periods. Values in black color represents the mean rainfall anomalies averaged over India. Improvement of mean rainfall after DBC is shown in %. Insignificant (< 95% confidence level) regions are masked with “ + ”. (Figure created using the Matlab R2023a; https://in.mathworks.com/).

**Figure 5:**
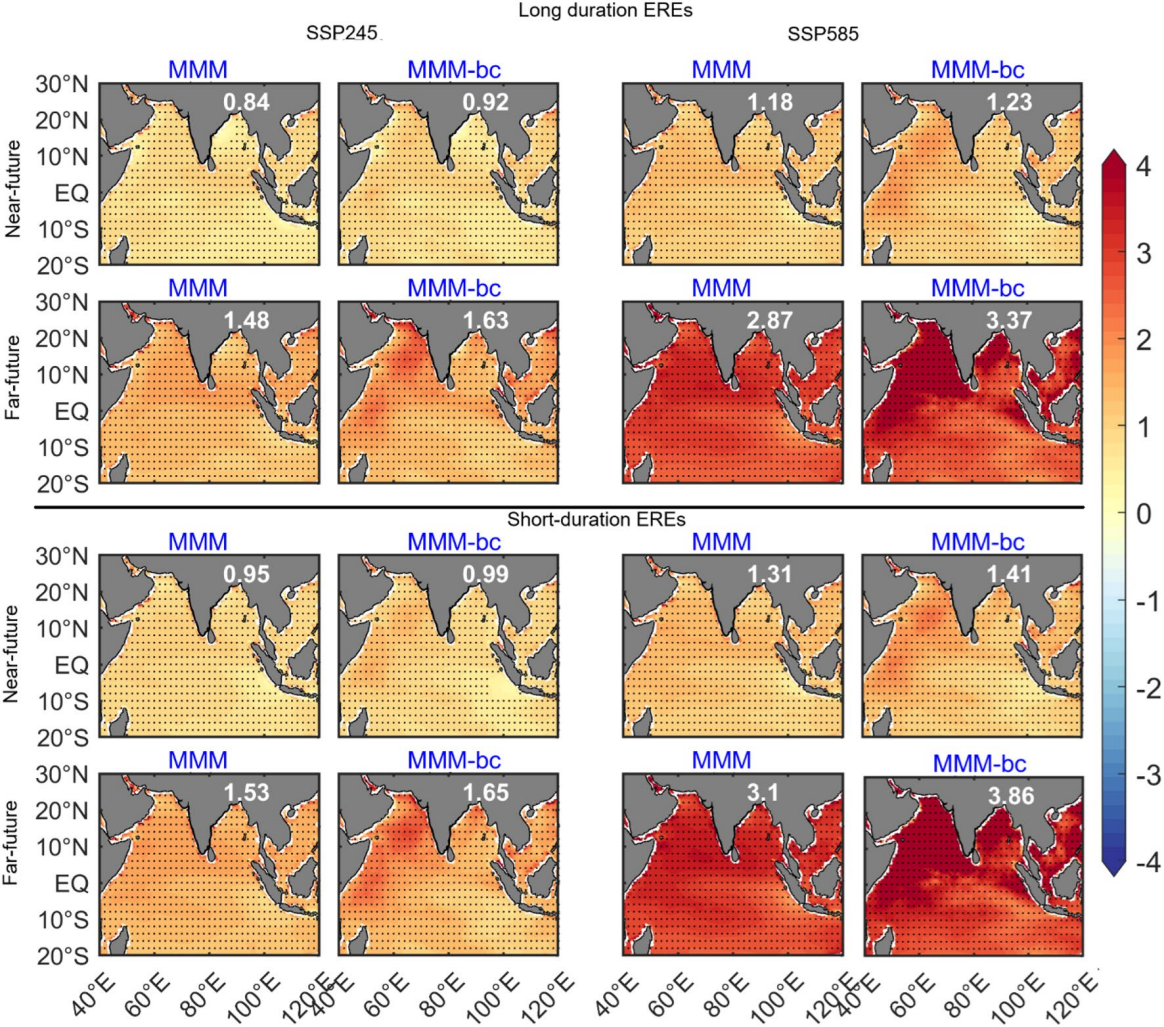
Composite of SST anomalies (w.r.to baseline period climatology) during long duration and short duration of EREs for SSP2-4.5 (SSP245), and SSP5-8.5 (SSP585) scenarios during near future and far future periods. Values in white color represents the mean SST anomalies averaged over the Indian Ocean. Significant (> 95% confidence level) regions are masked with “.”. (Figure created using the Matlab R2023a; https://in.mathworks.com/).

The composite of EREs precipitation under SSP245 and SSP585 scenarios is shown in Fig. 4. The composite of long-duration EREs in the near future under the SSP245 scenario shows an increase of rainfall over India and is about 0.74 (6.02) mm/day compared to the baseline EREs before (after) DBC. Moreover, after DBC, the rainfall distribution is widespread and this rainfall distribution and intensity shows 78% improvement. Under the SSP585 scenario, a 10% rainfall increase is found in the near future over India after DBC and is associated with the long duration EREs. The composite of long-duration EREs in the far future clearly shows an increase of rainfall by ~ 24% (65%) under the SSP245 (SSP585) scenario compared to the near future EREs after DBC. Significant improvement in rainfall intensity and distribution is seen after DBC. Under the SSP585 scenario in the far future, the intense convective rainfall is situated in the monsoon core region extends from west to east India, and Western Ghats. The mean rainfall over India during the short duration EREs is relatively less compared to long duration EREs. The rainfall distribution over India is significantly improved in the short duration EREs after DBC, whereas peak intensity of rainfall is noticed in the far future short duration EREs over west India under the SSP585 scenario. We have validated the DBC products of JJAS mean rainfall, R95 thresholds, rainfall distribution associated with the long and short duration of EREs (figure not shown) with the Tropical Rainfall Measuring Mission (TRMM) satellite measurement of precipitation. Results obtained with TRMM data are comparable with IMD data in seasonal mean rainfall and R95 thresholds over India. The composite of SST anomalies during EREs under the SSP245 and SSP585 scenarios is shown in Fig. 5. It is seen that warming rates of SST is significantly high in all EREs composites compared to the baseline. This SST warming is ~ 0.62 °C (~ 0.73 °C) high in the far future EREs compared to the near future EREs before (after) DBC in the SSP245 scenario. Further, the SST warming rates associated with the EREs are high in the SSP585 scenario. It is found that the SST warming is ~ 1.67 °C (~ 2.12 °C) in the far future EREs before (after) DBC. Further, SST warming is high in the short duration EREs compared to the long duration EREs, under the SSP585 scenario in the far future EREs. Analysis shows that the warming rates of SSTs associated with the EREs significantly improved after DBC.

**Figure 6:**
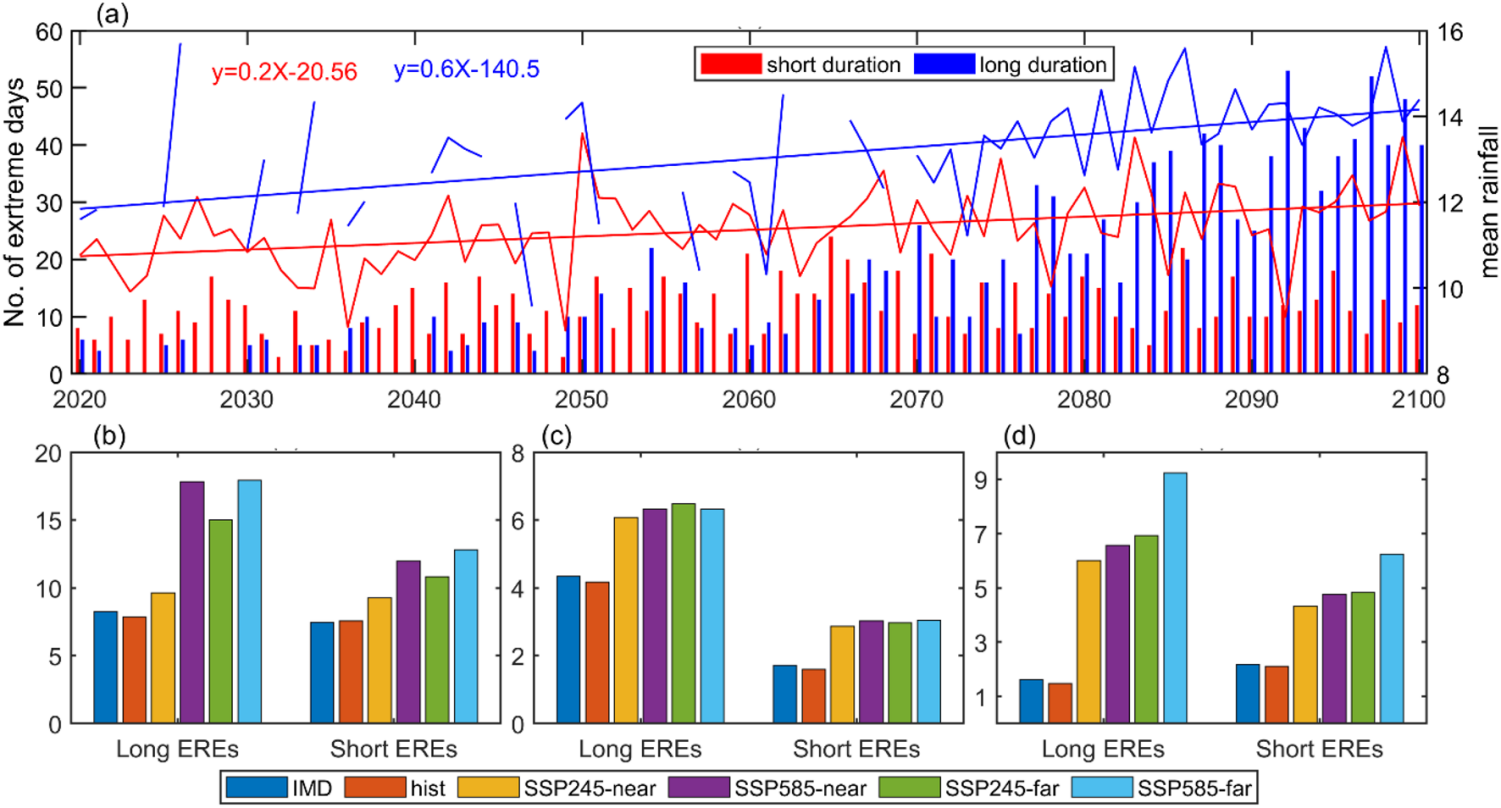
(**a**) Number of short and long duration EREs over monsoon core region during JJAS season (bars) and associated mean rainfall over India (line) for high emission scenario (SSP5-8.5) of MMM-bc product. The equation in red and blue color represents the trend of short and long duration of EREs for the period 2020–2100, respectively. (**b**) Area (%) experiencing the twofold of rainfall exceeding the mean rainfall over India associated with the EREs for the base period. (**c**) duration of ERES over monsoon core region. (**d**) All India mean rainfall associated with the EREs over monsoon core region. (Figure created using the Matlab R2023a; https://in.mathworks.com/).

The frequency of short and long duration EREs for the SSP585 scenario is shown in Fig. 6a. It is found that, before 2060, the number of short duration EREs are high compared to long duration EREs, however after 2060, long duration EREs are increasing compared to the short duration EREs over the monsoon core region. It is also found that, the mean rainfall associated with the short duration EREs is higher than long duration EREs over India. In the observations, ~ 8% of Indian land experiences extremely heavy rainfall with a long duration of EREs, however in the future scenario, this area is likely to increase by ~ 18% (Fig. 6b). Consistent with the long duration of EREs, extreme rainfall area associated with the short duration EREs also increasing in the future (Fig. 6c). In the historical period, all India mean rainfall associated with the short duration EREs is high compared to the long duration EREs, however, in the future scenario, MMM-bc product shows increase of mean rainfall associated with the long duration ERE and is relatively high compared to the short duration EREs (Fig. 6d). The analysis of Total Number of Days per year (TND) and Frequency of Occurrence (FoO) for short and long duration of EREs over India in the observations and CMIP6 models reveals that, TND is ~ 4 (~ 8) for long (short) duration of EREs in the observations, however, MMM highly overestimates the TND and FoO over India. On the other hand, MMM-bc shows consistency in TND days when compared to the observations. Altogether, under SSP585 scenario, in the near future MMM-bc shows the increase of long and short duration of EREs by about 5 and 8 days, whereas, in the far future long duration EREs are increasing (9 days) and short duration of EREs are decreasing (5 days) (Fig. S8).

## Discussion

Future precipitation and SST changes are critical constraints on developments and the socio-economic status of developing countries. As a consequence of global warming, the trends in precipitation and SST are expected to steeply increase, and this projection is particularly crucial for countries like India^48^. Estimating future changes of EREs and its associated SST variability is important to assess the mitigation and adaptation options within a sustainable development framework. Considering this, we study the spatio-temporal variability of EREs and its association with SST over the Indian summer monsoon region based on state-of-the-art seventeen CMIP6 models.

To estimate future precipitation changes in the twenty-first century, we first downscaled and bias corrected CMIP6 products (precipitation and SST) against observations on to a high-resolution (0.25°) for both the historical period and projections. Historical simulations and projections from SSP245 and SSP585 are used to estimate EREs and its associated SST variability for the baseline period (1982 to 2014) and the near future (2031–2060) and far future (2071–2100).

The results are summarized as follows.

- For the present-day period, the estimated seasonal mean rainfall and SST are strongly underestimated in CMIP6 simulations before downscaled and bias corrected (DBC). On the other hand, noticeable improvement in seasonal mean rainfall is seen in DBC product. Seasonal mean rainfall is expected to be 14(18)% and 20(35)% more in the near (far) future compared to the baseline period for SSP245 and SSP585, respectively based on the DBC product.
- In the baseline period, after DBC (6 mm/day), the scaling rates are consistent with the observations (7 mm/day). However, before DBC, MMM showed a peak intensity of precipitation, which is about 3 mm/day. A significant decreasing trend in the magnitude of scaling rates from the baseline period is seen in the SSP245 and SSP585 scenarios. The observations show SST peak at ~ 28.5 °C, whereas it is at ~ 27.7 °C in MMM, and at ~ 28.5 °C in MMM-bc. This analysis suggests that the DBC products of precipitation and SST well captured the scaling rates during monsoon season.
- The mean R95 precipitation values over India are about 48.6 mm/day according to IMD rainfall; however, before DBC, it was about 25.8 mm/day. After DBC, it improved by 66%, which is consistent with the observation. In the far future, it is projected to increase by 6% and 21%, respectively, under SSP245 and SSP585 scenarios.
- The composite precipitation anomalies for long and short-duration EREs for the baseline period showed intense precipitation over monsoon trough and Western Ghats in the observations. However, CMIP6 simulations strongly underestimate the intensity and spatial distribution of rainfall associated with the EREs. After DBC, the rainfall distribution and intensity improved by 96(87)% for long(short) duration EREs over central India. At the same time, the composite of SST associated EREs shows cooling over the WIO and north BoB and warming over the east equatorial Indian Ocean (EEIO) for the baseline period in the observations. In case of MMM based composite SST in the EEIO showed weak warm SST in the EEIO, whereas in MMM-bc, SST in the EEIO is consistent with the observations.
- The composite of long-duration EREs in the far future clearly shows the rainfall increase by ~ 26% (65%) under the SSP245 (SSP585) scenario compared to the near future EREs after DBC. Similarly, Indian Ocean SST warming is ~ 0.62 °C (~ 0.73 °C) high in the far future long duration EREs compared to the near future EREs before (after) DBC in the SSP245 scenario. Further, the SST warming is ~ 1.67 °C (~ 2.12 °C) high in the far future EREs before (after) DBC.
- It is also found that, before 2060, the number of short duration EREs is high compared to the long duration EREs, and vice versa after 2060 (which is a two-fold increase of long duration EREs compared to the short duration EREs over the monsoon core region). The mean rainfall associated with the long duration EREs is higher than short duration EREs over India under higher emission scenarios.
- This study provides DBC precipitation and SST products for the historical periods and future projections, results recommend the establishment of national climate change adaptation policies in general as well as to be specific for the EREs.

## Methods

To study the projected changes in EREs over India, we have used the precipitation and SST from 17 GCMs (Table S1) of the CMIP6 group, in which daily data is available. The precipitation and SST are downscaled to 0.25°x0.25°, and the bias is corrected by Empirical Quantile Mapping (EQM)^49^. The reference observed rainfall datasets and SST over the Indian Ocean used for developing these downscaled and bias corrected datasets are from the India Meteorological Department (IMD^50^) and the National Oceanic and Atmospheric Administration (NOAA). Climate change studies need bias-corrected projections for effective decision-making at regional and local scales^41^. Downscaling and bias correction techniques are the two methods that are usually used to get relatively accurate climate change projections at a regional scale^49^. More details of downscaling and bias correction is provided in the previous study^51^. High-resolution data products are important to obtain consistent climate projections at a local or provincial scale, which is essential for framing adaptation strategies^52^. Suppose the bias corrected dataset is consistent with observation for a climatological mean period, in that case, conclusions on the projected changes and their implications in different sectors (e.g., water resources and agriculture) are more acceptable. In this study, the entire analysis is carried out for the baseline period (1980–2014), near future (2031–2060), and far future (2071–2100)^53^ for CMIP6 models before and after downscaling and bias corrected data.

Each model’s historical run period from 1980 to 2014 is forced to simulate the future projections of 2015 to 2100 under SSP2-4.5 (SSP245) and SSP5-8.5 (SSP585) emission scenarios. Though five SSPs are simulated by keeping the different radiative forcing estimates by the end of the twenty-first century^35^, we considered SSP245 and SSP585 to comprehend future climate scenarios. The SSP245 is selected due to its central position in the key metrics of the mitigation and adaptation challenges^54^, and the SSP585 is chosen as it is characterized by the high and low challenges for mitigation and adaptation, respectively. Observed rainfall datasets of IMD version 4 (IMD4) with 0.25° × 0.25° grid resolution^50^ and SST from NOAA OI v2 are used to evaluate the model skill for 1980–2014, SST from 1982 to 2014. Further, we adopted different metrics to estimate the relative changes in the future compared to the baseline period. The present study focuses on the EREs over the monsoon core region (18 to 28^°^N and 65 to 88^°^E)^55,56^ during the ISM. However, the selected region in this study is dominated by passage of synoptic scale disturbances from the BoB, which mainly contribute to the EREs over the monsoon core region and the seasonal mean rainfall over this region and several recent studies have highlighted this region to identify EREs^51,55,56^. An ERE at each grid is identified using the 95 percentile threshold (R95). Any grid point exceeding the threshold (R95) of precipitation is termed an ERE at that grid point^57^. The rainfall events over an area > 30,000 km^2^ are considered and are further classified into long duration (> 4 days) and short duration (≤ 4 days)^58^. While selecting the EREs, we adopted the iterative grid point method, in which consecutive grid points of the days that exceed the R95 threshold are only considered for analysis. It mainly takes the frequency of occurrence and synoptic signatures into account. The frequency and threshold of EREs are calculated based on the R95 at each grid point during the Indian summer monsoon. Note that we have used observed thresholds to identify the EREs in the observations, and to identify the EREs in the models, we have considered the model-specified thresholds of R95. Improving the mean percentage would provide an opportunity to better utilization of developed data sets. Under the global warming scenario, the frequency and duration (from hours to days) of extreme rainfall events are increasing alarmingly^59^. This is the rationale of choosing short and long duration extreme rainfall events is understanding the distribution of rainfall and SST associated with EREs, and their projected changes in the warming climate are important and provides valuable information. The holistic approaches used in the present study would be useful to understand spatial and temporal variations of the EREs and mitigate the impacts of extreme events in a changing climate. Process that governs the EREs over India and its variability is left for the future study, because it requires other atmospheric downscaled and bias corrected parameters to draw much more scientific conclusions.

### Downscaling and *bias* correction

The present study utilizes the Empirical Quantile Mapping (EQM)^49^ approach to bias-correct the daily precipitation hindcasts with a spatial resolution of 0.25° over the Indian summer monsoon region. The spatial resolution of the models varies from about 63 km to 150 km. Models precipitation and SST data are re-gridded to 0.25° to maintain consistency among them and to better representation in the elevated surfaces. In this study we have used the bilinear interpolation method^41,51^. The interpolation method used in this study does not significantly impact the spatial dissemination of climatology, biases, or annual cycle. In order to produce reliable estimates of regional and local climate impact assessment, the systematic biases in the models are then corrected using EQM^41^. The quantile mapping method adjusts the variability with the observed variability using a transfer function, which could be parametric or nonparametric. The transfer function is formulated as in Mishra et al.^41^ and Konda et.^51^, which is given below.

$${X}_{m}^{o}=f({X}_{m})$$

where ${X}_{m}^{o}$ is the bias-corrected model output. The transfer function for the known statistical distributions of ${X}_{m}$ and ${X}_{o}$ is

$${X}_{m}^{o}={F}_{0}^{-1}({F}_{m}{(X}_{m}))$$

${F}_{m}$ and ${F}_{0}$ are the cumulative distribution functions (CDFs) of ${X}_{m}$ and ${X}_{0}$ respectively.

As in Mishra et al.^41^ empirical CDFs in EQM are estimated from the percentiles calculated from ${X}_{m}$ and ${X}_{0}$. As a result, EQM and its variants are applied to the precipitation even if their underlying distributions are different. We have used the normalized root mean square error, standard deviation, correlation, absolute mean bias, percentage change in bias, and mean error etc. to quantify the skill of the models in representing the mean features.

### Supplementary Information

Supplementary Information.

## Data Availability

Downscaled products are available on request from G.N.K. and J.S.C. All other datasets used in this study are freely available for download from Earth System Grid. Federation (ESGF; https://esgf-node.llnl.gov/projects/cmip6/), IMD, and NOAA.

## Code availability

The data in this study is analyzed with Matlab R2023a (https://in.mathworks.com/). The relevant codes in this work are available, upon request, from the corresponding author GNK.
