# Supplementary material for: Temporal and spatial aggregation of rainfall extremes over India under anthropogenic warming
Source: Sci Rep. 2024 May 31;14:12538. doi: 10.1038/s41598-024-63417-w (PMC11143250; doi:10.1038/s41598-024-63417-w)
Supplement: Supplementary file 1 — Supplementary Information. [file 41598_2024_63417_MOESM1_ESM.docx]

**Temporal and spatial aggregation of rainfall extremes over India under anthropogenic warming**

**Gopinadh Konda^1*^, Jasti S. Chowdary^1^, C. Gnanaseelan^1^, Naresh Krishna Vissa^2^, and Anant Parekh^1^**

^1^Indian Institute of Tropical Meteorology, Ministry of Earth Sciences, Pune-411008, India

^2^ Department of Earth and Atmospheric Sciences, National Institute of Technology Rourkela-769008, India

*gopinadh.konda@tropmet.res.in

**Supplementary Information:**


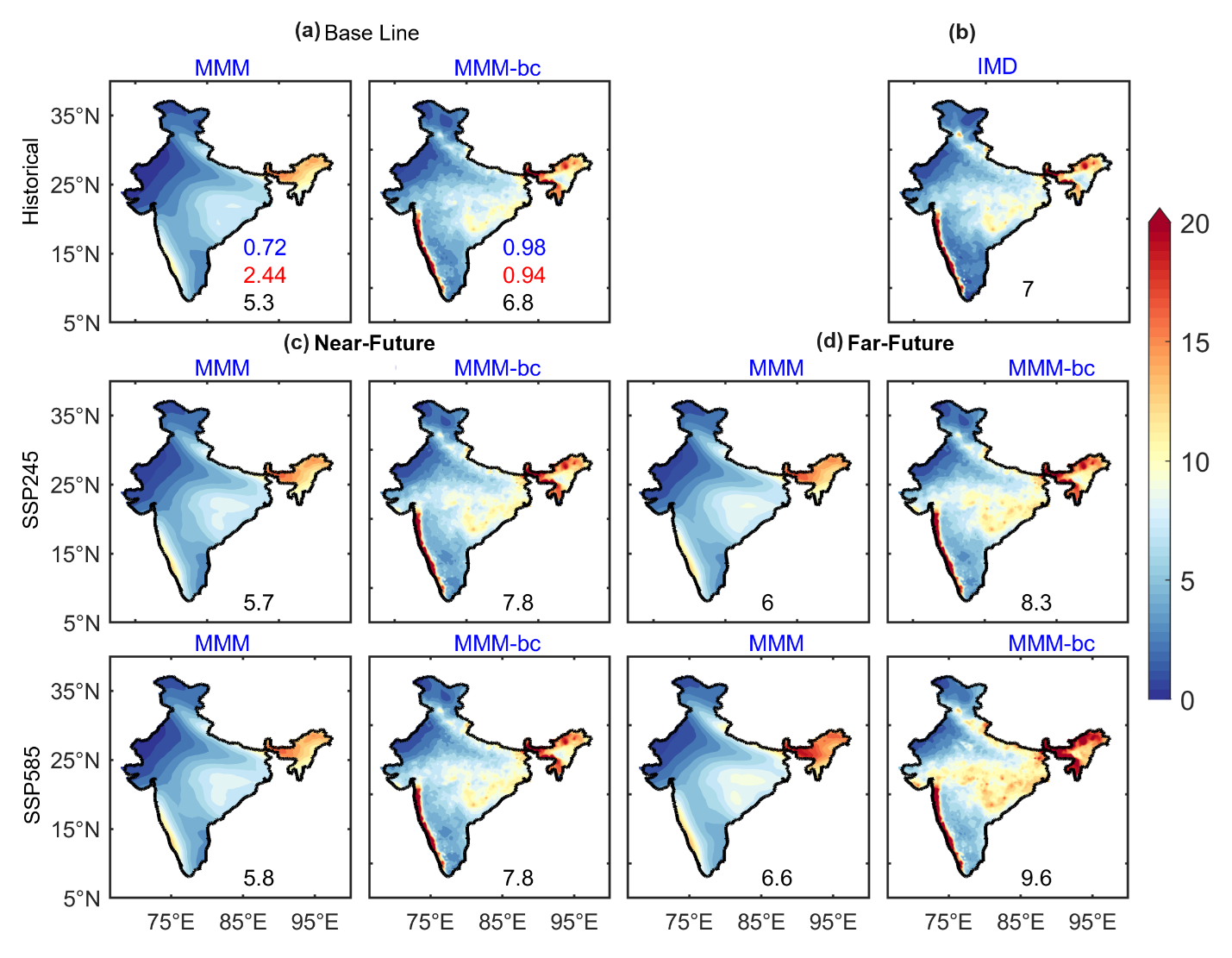


Figure S1: Multi model mean of Indian summer monsoon (JJAS) seasonal mean rainfall (mm/day) over India for historical, SSP2-4.5 (SSP245), and SSP5-8.5 (SSP585) scenarios varying from baseline period (a; 1980-2014), near future (c; 2031-2060) and far future (d; 2071-2100). Before DBC (MMM) and after DBC (MMM-bc). Values in blue (red) color represents the pattern correlation (RMSE, mm/day). Values in black color represents the seasonal and areal mean rainfall (mm/day). (Figure created using the Matlab R2023a; https://in.mathworks.com/).


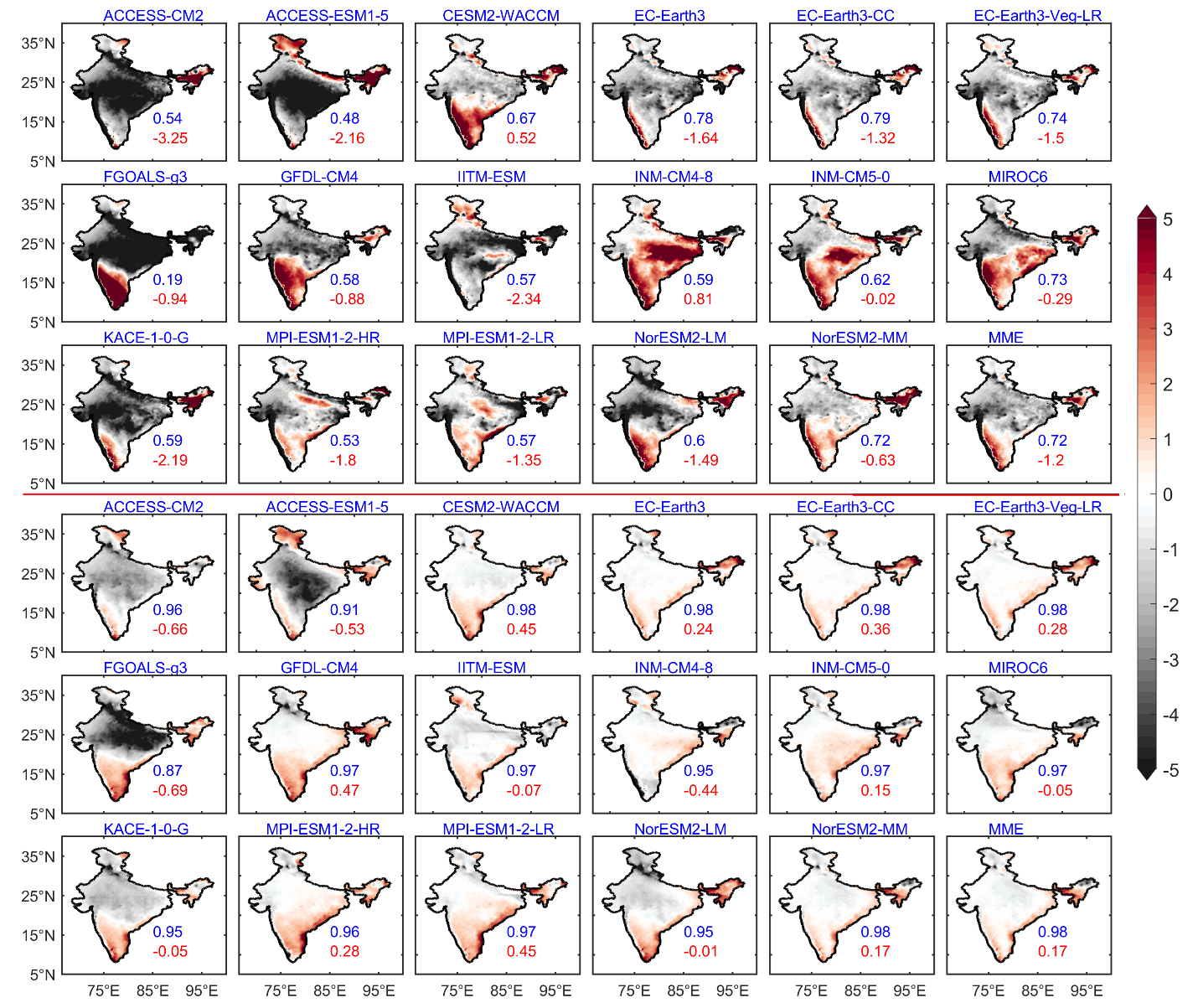


Figure S2: JJAS seasonal mean bias of rainfall for individual models and MME of the models, above black line for models before DBC, after DBC (below black line). Values in blue (red) color represents the pattern correlation (RMSE). (Figure created using the Matlab R2023a; https://in.mathworks.com/).


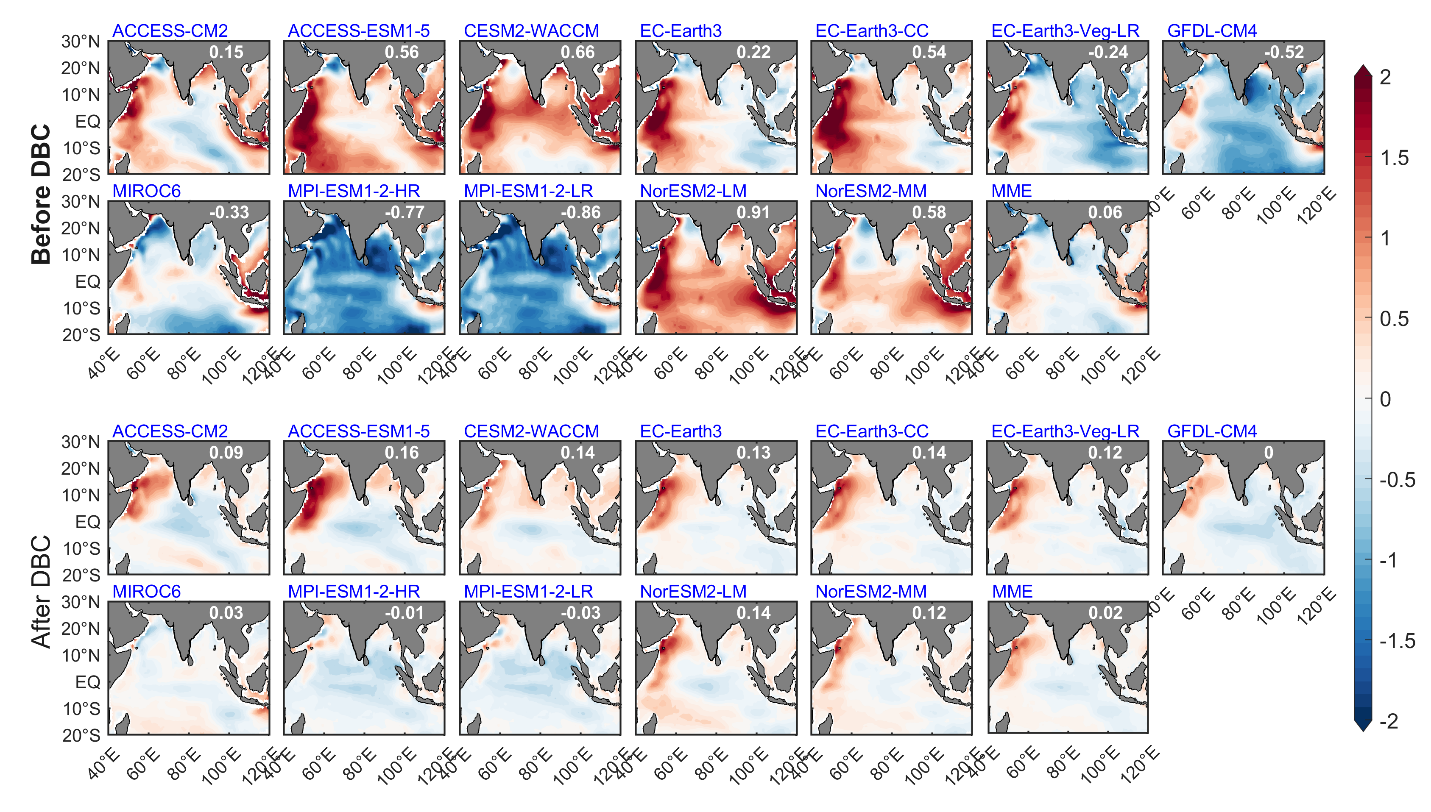


Figure S3: JJAS seasonal mean bias of SST (°C) for individual models for the historical period, top panel for before DBC, bottom panel for after DBC. Values in white color represents the mean bias (°C). (Figure created using the Matlab R2023a; https://in.mathworks.com/).


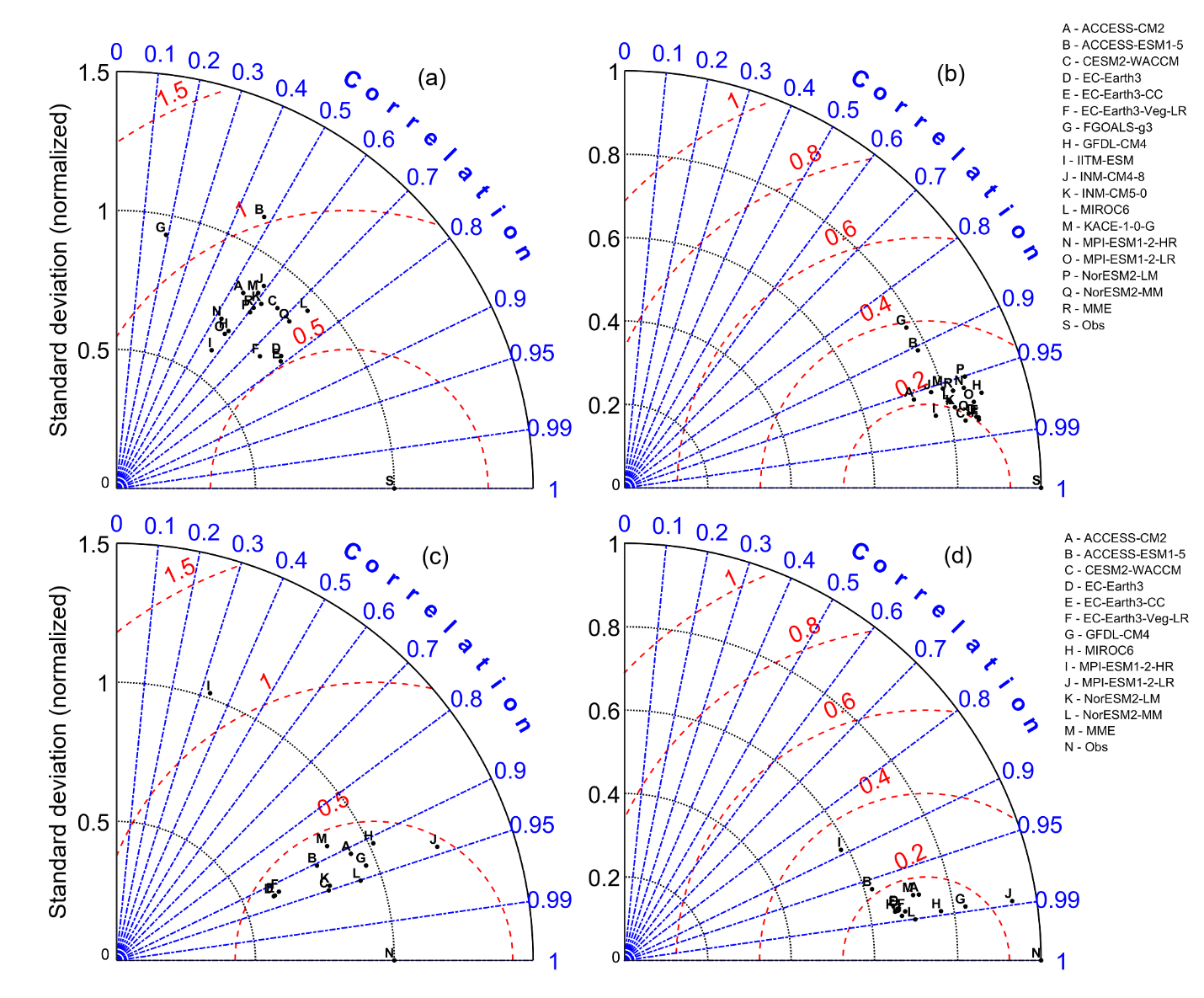
Figure S4: Taylor metric for ISM seasonal mean rainfall (over India) (a) for Before DBC, (b) for After DBC. (c) and (d) as of (a) and (b) but for SST (over Indian Ocean) for the baseline period. (Figure created using the Matlab R2023a; https://in.mathworks.com/).


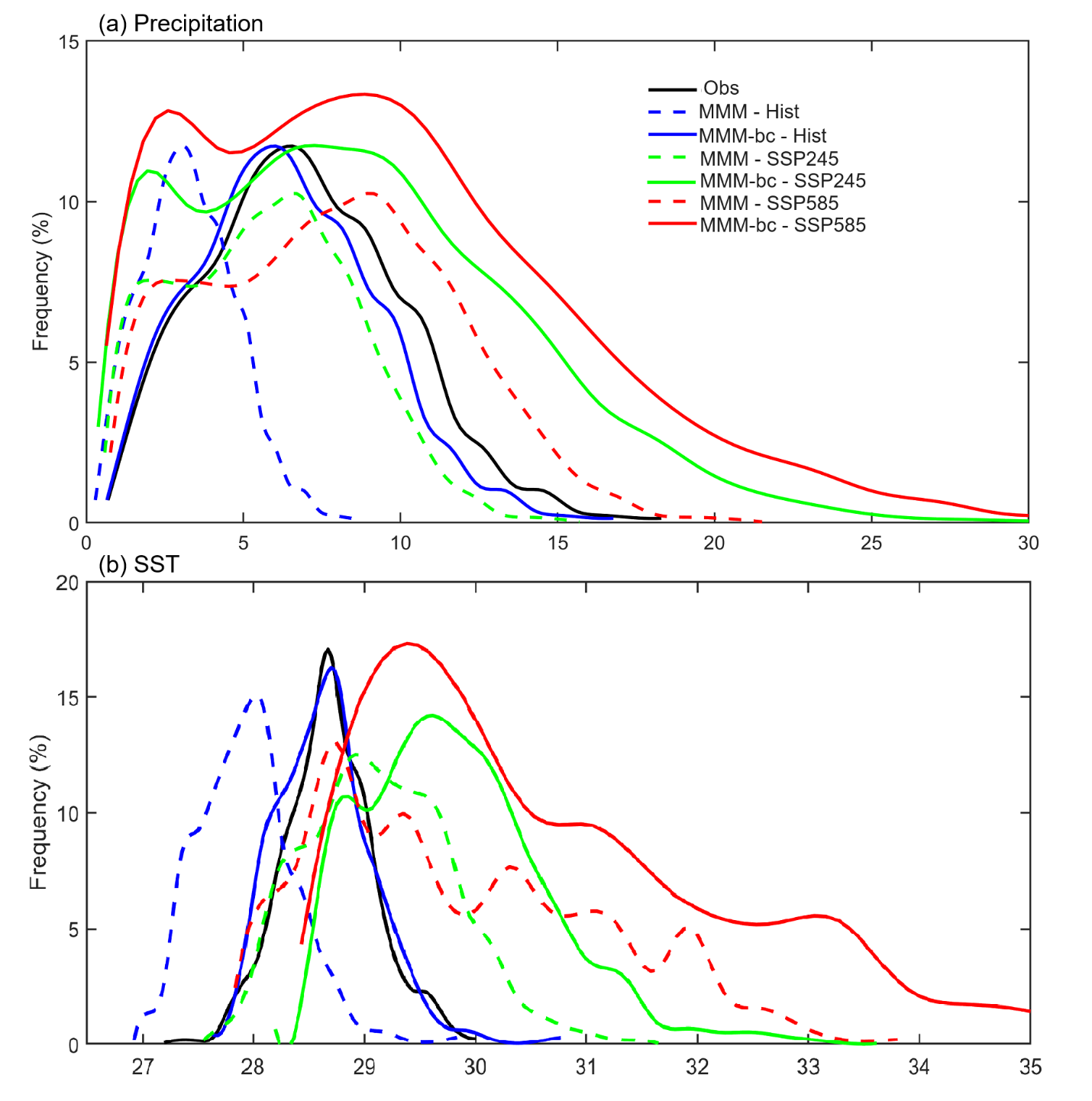
 Figure S5: Scaling/Frequency distribution of (a) precipitation (over India; X-axis in mm/day) and (b) SST (over Indian Ocean; X-axis in ^o^C). (Figure created using the Matlab R2023a; https://in.mathworks.com/).


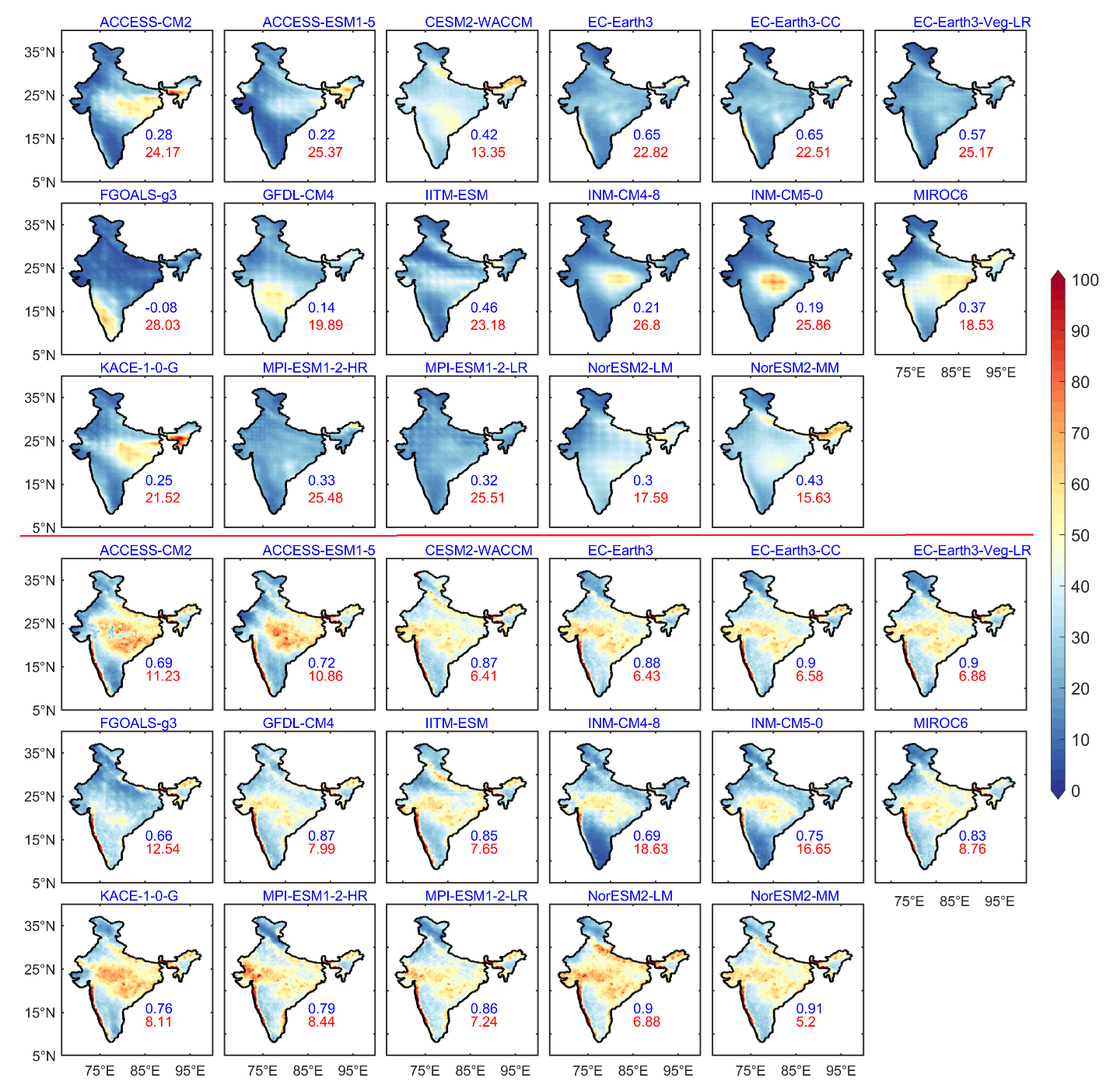


Figure S6: JJAS seasonal R95 thresholds for individual models, above black line for models before DBC, after DBC (below black line). Values in blue (red) color represents the pattern correlation (RMSE). (Figure created using the Matlab R2023a; https://in.mathworks.com/).


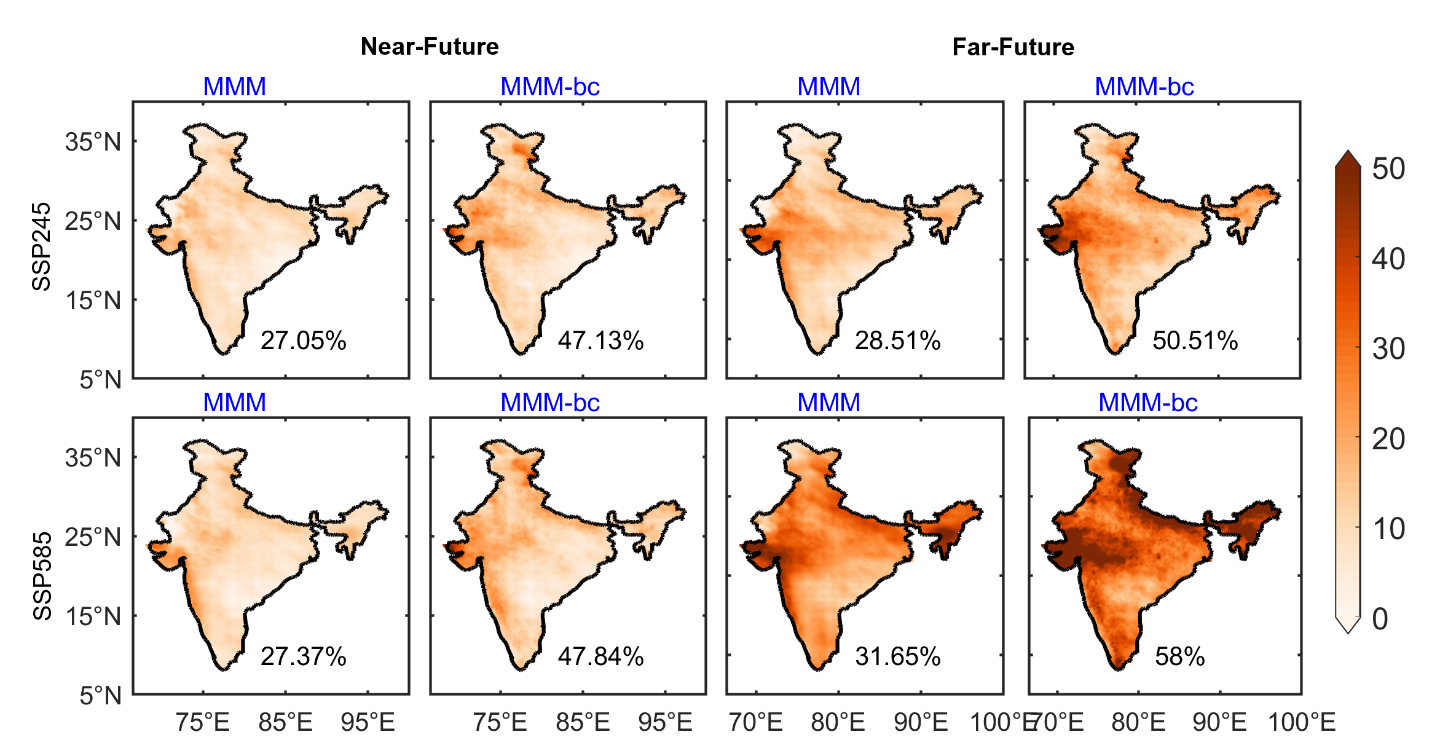


Figure S7: Percentage change of R95 threshold over India for near future and far future under SSP2-4.5 (SSP245) and SSP5-8.5 (SSP585) scenarions for before DBC (MMM), and after DBC (MMM-bc). Values in black color represents the mean percentage change. Percentage change is calculated w.r.to the baseline period. (Figure created using the Matlab R2023a; https://in.mathworks.com/).


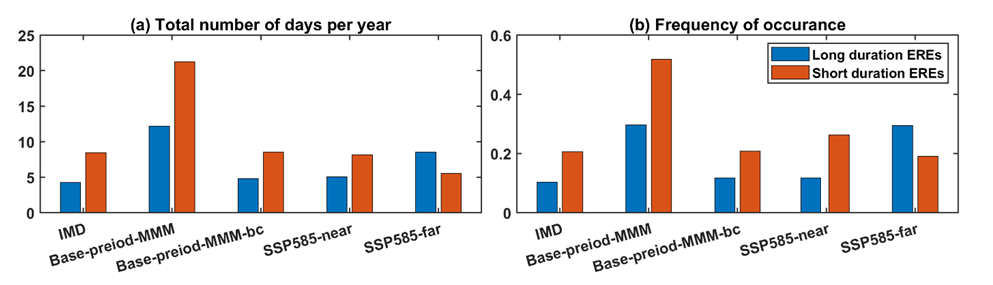


Figure S8: Long and short duration of EREs computed from IMD, MMM, and MMM-bc for baseline period, and future projection (SSP585). The average Total number of days per year, Frequency of occurrence of long and short duration EREs presented in (a) and (b), respectively. (Figure created using the Matlab R2023a; https://in.mathworks.com/).

Table S1: Minutiae of the 17 CMIP6 Models, that joined in the CMIP6 project.

| **Source ID** | **Atmospheric Component**  **(lon x lat; vertical levels )** | **Oceanic Component**  **(lon x lat; depth levels )** |
| --- | --- | --- |
| ACCESS-CM2 | MetUM-HadGEM3-GA7.1 (92/144; 85) | ACCESS-OM2 (GFDL-MOM5; 360/300; 50) |
| ACCESS-ESM1-5 | HadGAM2 (192 x 145; 38) | ACCESS-OM2 (GFDL-MOM5; 360/300; 50) |
| CESM2-WACCM | WACCM6 (288/192; 70) | POP2 ( 320/384; 60) |
| EC-Earth3 | IFS cy36r4 (512/256; 91) | Nemo 3.6 ( 362/294; 75) |
| EC-Earth3-CC | FS cy36r4 (320 x 160; 91) | NEMO3.6 (362 x 292; 75) |
| EC-Earth3-Veg-LR | FS cy36r4 (320 x 160; 62) | NEMO3.6 (362 x 292; 75) |
| GFDL-CM4 | GFDL-AM4.0.1 ( 360/180; 33) | GFDL-OM4p25 (GFDL-MOM6, 1440/1080; 75) |
| IITM-ESM | IITM-GFSv1 (192 x 94; 64) | MOM4p1 (360 x 200; 50) |
| INM-CM4-8 | INM-AM4-8 (180/120; 21) | INM-OM5; 40 levels (sigma vertical coordinate) |
| INM-CM5-0 | INM-AM5-0 (180/120; 73) | INM-OM5; 40 levels (sigma vertical coordinate) |
| KACE-1-0-G | MetUM-HadGEM3-GA7.1 (192/144; 85 ) | MOM4p1 ( 360/200; 50) |
| MIROC6 | CCSR AGCM (256/128; 81) | COCO4.9 ( 360/256; 63) |
| MPI-ESM1-2-HR | ECHAM6.3 ( 384/192; 95) | MPIOM1.63 ( 802/404; 40) |
| MPI-ESM1-2-LR | ECHAM6.3 (192 x 96; 47) | MPIOM1.63 (256 x 220; 40) |
| NorESM2-LM | CAM-OSLO (144/96; 32 ) | MICOM ( 360/384; 70) |
| NorESM2-MM | CAM-OSLO (288/192; 32 ) | MICOM ( 360/384; 70) |
